# Phylogeography of the Coastal Mosquito *Aedes togoi* across Climatic Zones: Testing an Anthropogenic Dispersal Hypothesis

**DOI:** 10.1371/journal.pone.0131230

**Published:** 2015-06-24

**Authors:** Teiji Sota, Peter Belton, Michelle Tseng, Hoi Sen Yong, Motoyoshi Mogi

**Affiliations:** 1 Department of Zoology, Graduate School of Science, Kyoto University, Kyoto, 606–8502, Japan; 2 Department of Biological Sciences, Simon Fraser University, Burnaby, BC V5A 1S6, Canada; 3 Department of Zoology, The University of British Columbia, Vancouver, BC, V6T 1Z4, Canada; 4 Institute of Biological Sciences, University of Malaya, Kuala Lumpur, 50603, Malaysia; 5 Division of Parasitology, Department of Pathology and Biodefense, Faculty of Medicine, Saga University, Saga, 849–8501, Japan; St. Petersburg Pasteur Institute, RUSSIAN FEDERATION

## Abstract

The coastal mosquito *Aedes togoi* occurs more or less continuously from subarctic to subtropic zones along the coasts of the Japanese islands and the East Asian mainland. It occurs also in tropical Southeast Asia and the North American Pacific coast, and the populations there are thought to have been introduced from Japan by ship. To test this hypothesis, the genetic divergence among geographic populations of *A*. *togoi* was studied using one mitochondrial and three nuclear gene sequences. We detected 71 mitochondrial haplotypes forming four lineages, with high nucleotide diversity around temperate Japan and declining towards peripheral ranges. The major lineage (L1) comprised 57 haplotypes from temperate and subarctic zones in Japan and Southeast Asia including southern China and Taiwan. Two other lineages were found from subtropical islands (L3) and a subarctic area (L4) of Japan. The Canadian population showed one unique haplotype (L2) diverged from the other lineages. In the combined nuclear gene tree, individuals with mitochondrial L4 haplotypes diverged from those with the other mitochondrial haplotypes L1—L3; although individuals with L1—L3 haplotypes showed shallow divergences in the nuclear gene sequences, individuals from Southeast Asia and Canada each formed a monophyletic group. Overall, the genetic composition of the Southeast Asian populations was closely related to that of temperate Japanese populations, suggesting recent gene flow between these regions. The Canadian population might have originated from anthropogenic introduction from somewhere in Asia, but the possibility that it could have spread across the Beringian land bridge cannot be ruled out.

## Introduction

Major human disease vectors of the mosquito tribe Aedini (Culicidae: Culicinae) including the yellow fever mosquito *Aedes aegypti* and the Asian tiger mosquito *A*. *albopictus* are well known examples of anthropogenic dispersal across the world [1–4]. A recent example is the spread of *Aedes japonicus* to the United States, Canada and European countries where it was probably introduced in used tires from Japan [5–6]. Many species of Aedini breed in small water bodies including artificial containers, lay desiccation and freeze resistant eggs, and hence have potential to be transported by ships as eggs or larvae in water tanks or used tires. Finding the origin and means of dispersal of disease-vectors spreading across the world is crucial to predicting the risk of vector colonization and implementing appropriate control programs.

Anthropogenic dispersal may have resulted in the wide distribution across climatic zones in the aedine species *Aedes togoi*, which is widely distributed from subtropical to subarctic East Asia, breeding in coastal rock pools as well as artificial containers [7]. This species is a known vector of human and canine filariasis, and Japanese encephalitis virus has been isolated from field-collected mosquitoes [7–10]. The occurrence of *A*. *togoi* has also been reported from tropical Southeast Asia [11–13] and the Pacific coast of Canada and the USA [14–16]. These peripheral populations were discovered in the late 20th century and may have been introduced from Japan by ships. In the temperate zone, this species has several generations per year, overwinters as diapausing larvae or eggs and shows facultative autogeny (egg production without blood feeding) [17–18]. Over their geographic range, *A*. *togoi* populations differ in life history traits such as body size, larval diapause (delayed development in response to short-day condition) and autogeny [19–22]. The Canadian population has life-history traits similar to those of temperate Japanese populations [20–24], consistent with the introduction hypothesis. However, tropical Asian populations show traits different from temperate ones: small body size, obligatory autogeny and lack of larval diapause [19–22]. This finding raises doubts about the introduction hypothesis [19]. The genetic relationships among different populations might resolve the origins of the peripheral *A*. *togoi* populations. In this study, we therefore studied geographic genetic variations in *A*. *togoi* using mitochondrial and nuclear gene sequences to investigate the validity of the anthropogenic introduction hypothesis.

## Materials and Methods

### Sampling and DNA extraction

**Fig 1:**
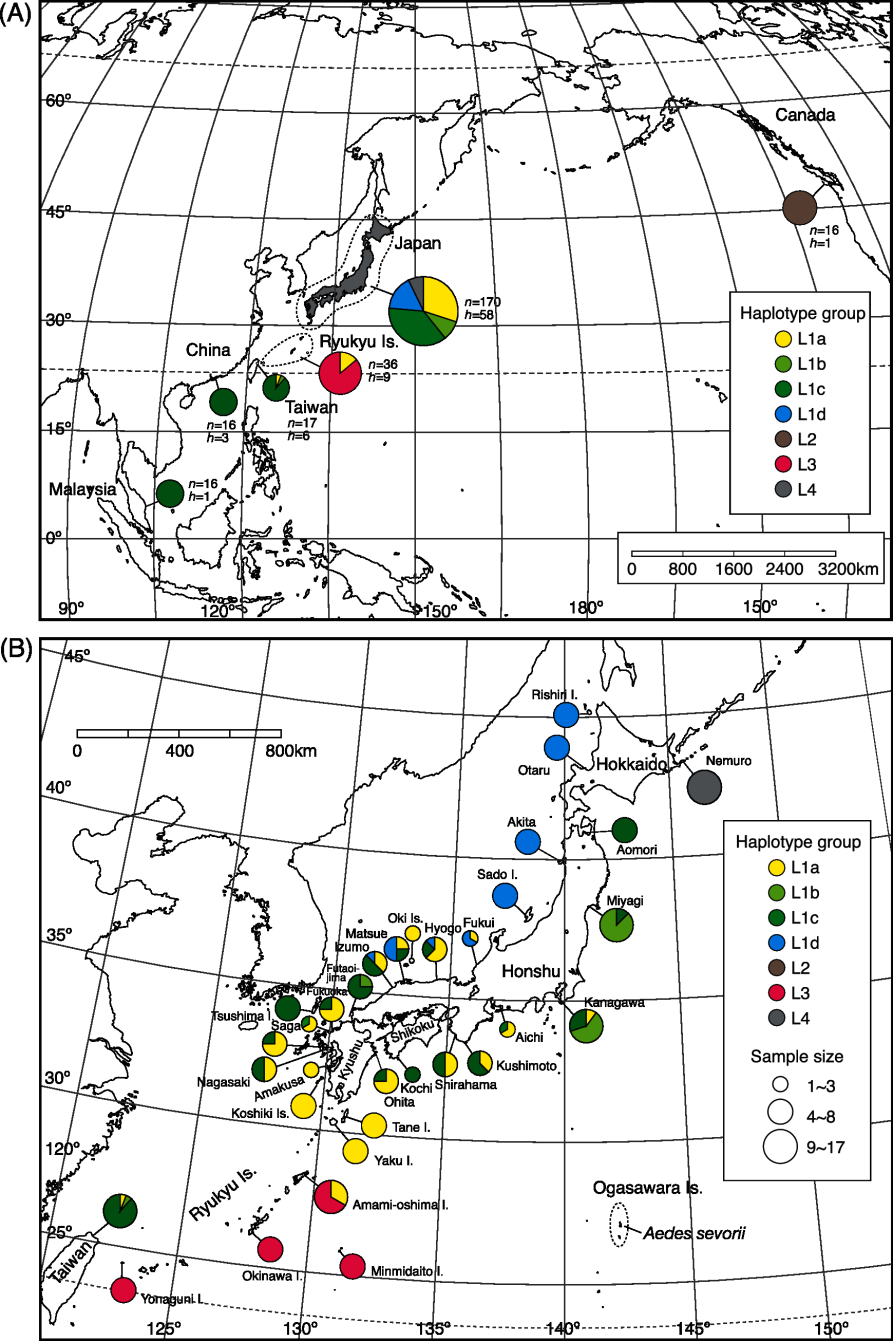
Sample localities of *Aedes togoi* with mitochondrial COI haplotype composition. (A) Sample localities in Asia and Canada. Sample size (*n*) and the number of haplotypes (*h*) for each locality are indicated. (B) Localities in Japan, with one locality in Taiwan.

This study did not include protected or endangered species, and no permission was required to collect the study species, *Aedes togoi* and *Aedes savoryi*. All the collection sites were in unprotected areas accessible to the public, and permission from landowners was obtained whenever necessary. A total of 282 larvae or adults of *A*. *togoi* were collected at 42 field sites (S1 Table; Fig 1). Larval and adult specimens were fixed in 95–99% ethanol and stored in a refrigerator until DNA was extracted. Total genomic DNA was extracted from individual larvae or adults using a Wizard DNA purification kit (Promega). We also used adult specimens (preserved in ethanol) from 3 old laboratory colonies originating in Taiwan, Malaysia and Thailand (S1 Table). We used 8 larvae of *A*. *savoryi* from Ogasawara (Bonin) Islands, Japan, as the outgroup (S1 Table). *Aedes savoryi* is morphologically similar to *A*. *togoi* and considered as the sister species [25]. We examined the sister relationship based on three nuclear gene sequences from 12 culicine species including *A*. *togoi* and *A*. *savoryi* (see S2 Table and S1 Fig for methods).

### DNA sequencing

We used partial sequences of the mitochondrial cytochrome oxidase subunit I (COI) gene and nuclear 28S rRNA (28S), CAD and white genes for phylogenetic analysis. CAD and white genes were selected based on a previously published mosquito molecular phylogeny [26]. A partial COI gene region was amplified using primers C1-J-2195: 5’-TTG ATT TTT TGG TCA TCC AGA AGT-3’ and TL2-N-3014: 5’-TCC AAT GCA CTA ATC TGC CAT ATT A-3’ [27]. Partial sequences of 28S rRNA, white, and CAD genes were amplified and sequenced using the following primers. For 28S, Dipt28SF: 5’-AGG GGA GGA AAA GAA ACT AAC AAG GA-3’ and Dipt28SR1: 5’-CTT GGT CCG TGT TTC AAG ACG GGT C-3’ [28]. For CAD, 819 bp exon sequence, CAD F1: 5’-GAA AGA CCC GGT GGA GTG CTG CTC ACC-3’; CAD R1: 5’-GCC ATC ACT TCA CCG ACA CTY TTC AT-3’. For white (825 bp aligned sequence encompassing 3 exon and two intron) white F1: 5’-TAY AAT CCG GCG GAY TTC TAC GTC CAA ATG-3’; white R1: 5’-ATC AGG AAY GGA ATA ATC ACC GGT GGR CCG-3’, and internal primers only for sequencing, white F2: 5’-CAA ACG GCG GTA AGA ATC AG-3’ and white R2: 5’-TGG AAG CTC AGC AGA AAA CA-3’. We redesigned the CAD and white primers with reference to the published sequences of these genes for some aedine species [26]. Sequence data are deposited in the DNA Data Bank of Japan (DDBJ: accessions LC025655—LC025878).

### Phylogenetic, population genetic and divergence time analysis

Alignment of sequences was done with MUSCLE version 3.831 [29]. Maximum-likelihood (ML) analysis was done with RAxML v8.0.0 [30] with rapid bootstrapping procedure using 1000 bootstrap analyses. We used partitioned analysis discriminating three codon positions, intron and rRNA sequence, and applied GTR+G substitution model to each partition because it is recommended as the most versatile model by the author of RAxML [30]. For COI and CAD gene sequences and the concatenated exon sequence of white gene, three codon positions were treated as different partitions. Each of the entire 28S gene sequence and the concatenated intron sequence of white gene was treated as one partition. A combined ML analysis was conducted for the nuclear gene sequences. We also constructed a phylogenetic network for the COI gene sequence to demonstrate a star-like topology of haplotype sequences using the neighbor-net algorithm in SplitsTree4 v4.13.1 [31]. Node credibility was assessed using 1000 bootstrap resampling. In addition, we constructed a population tree based on mean uncorrected p-distance between populations obtained using MEGA version 5.2.2 [32] and using the neighbor-joining method in PHYLIP version 3.69 [33]. To show latitudinal trend in genetic diversity, the nucleotide diversity of COI gene sequences at each locality was calculated using Arlequin version 3.5.1.3 [34].

The divergence times between COI haplotype lineages were estimated using a Bayesian relaxed clock model analysis implemented in BEAST version 1.80 [35]. For time calibration, we used disconnection of the Ryukyu Islands from the main islands of Japan (Kyushu, Shikoku and Honshu) 1.7 million years ago (mya). The Ryukyu Islands were separated from the East Asian mainland by tectonic movement and enlargement of the Okinawa trough during the late Pliocene and the early Pleistocene, and the land connection between the Ryukyu Islands and the main islands of Japan disappeared by 1.7 mya [36]. We assumed this geological event was related to divergence of the haplotype lineage of *A*. *togoi* specific to Ryukyu and set the node age prior to the branching of the Ryukyu haplotype lineage as a normal distribution with a mean of 1.7 my and a standard deviation of 0.17 my. In BEAST analysis, the COI sequences were partitioned by three codon positions and a GTR+G substitution model was used for each partition. We used an uncorrelated relaxed clock model with lognormal distribution of branch-specific rates, and the Markov Chain Monte Carlo (MCMC) run was performed for 10^8^ generations with a sampling frequency of 10^4^. The results were checked for effective sample sizes using Tracer version 1.6 [37]; and a consensus tree was obtained using TreeAnnotator version 1.8.0 in BEAST discarding the initial 1000-generation data as burn-in. Note that the calibration method we employed is uncertain for the relationship between the geological event and the haplotype divergence. However, we have also tested a divergence time estimate using a time-dependent evolutionary rate of insect mitochondrial gene sequences [38–39] and obtained divergence times that are almost within the 95% HPD (highest probability density) intervals of those obtained by the above analysis (see S3 Table).

## Results

**Fig 2:**
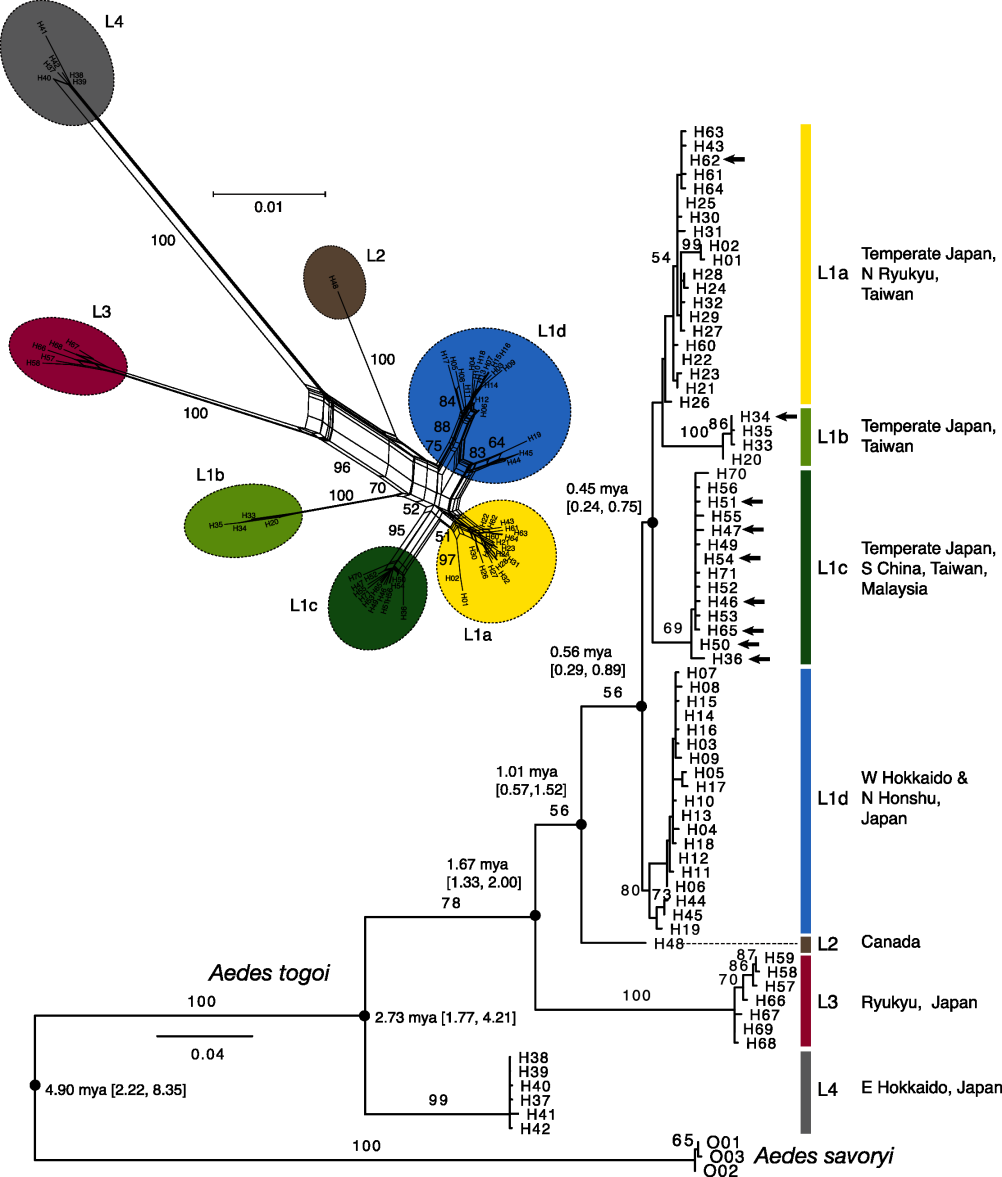
Maximum-likelihood tree and neighbor-net network of mitochondrial COI haplotypes. H-numbers (H001—H071) are haplotypes of *A*. *togoi*, and O001—O003 are those of *A*. *savoryi* (outgroup). In the ML tree, numerals on branches are bootstrap percentages (shown when >50%); arrows indicate haplotypes from Taiwan, southern China and Peninsular Malaysia, which occurred also in Japan except H34, H46 and H51; for nodes with closed circles, median node ages estimated by BEAST analysis are indicated with 95% HPD intervals in parentheses (see S3 Fig for details). In the network, numerals on branches are bootstrap percentages (shown when >50%).

**Fig 3:**
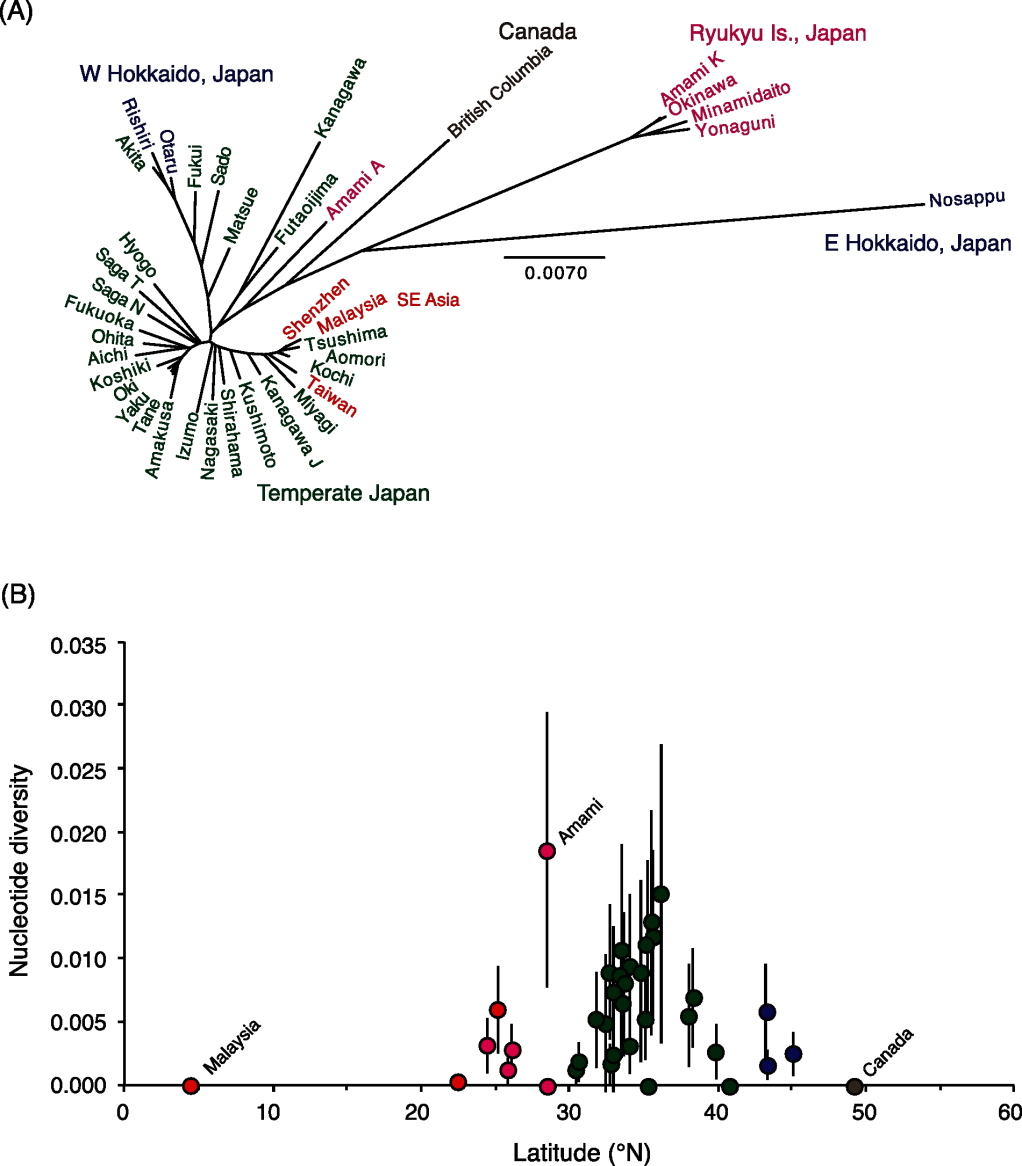
(A) Population tree constructed by neighbor-joining method based on mean uncorrected p-distance between populations. (B) Latitudinal change in the nucleotide diversity of COI sequences at each locality. Bars show ± SD. Open circles, Japanese islands except Ryukyu Islands; gray circles, Ryukyu Islands; black circles, outside Japan.

We confirmed the appropriateness of *A*. *savoryi* as the outgroup using three nuclear gene sequences (S2 Table; S1 Fig). We obtained an 817-bp COI gene sequence for 282 *A*. *togoi* and 8 *A*. *savoryi* specimens. We detected a total of 71 haplotypes for the mitochondrial COI sequence of *A*. *togoi* (Fig 2; S4 Table; DDBJ accessions LC025655—LC025758). The haplotypes were divided into four lineages L1—L4 (Fig 2), which had an estimated coalescent time of 2.7 mya (95% HPD interval, 1.8–4.2 mya; i.e. late Pliocene to early Pleistocene; see also S2 Fig). Of these lineages, L1 haplotypes were found from subarctic Hokkaido to subtropical Amami in Japan, highly diversified (57 haplotypes) and further clustered into four subgroups L1a—L1d, that comprised 20, 4, 14, and 19 haplotypes, respectively. These subgroups occurred in different regions; L1d occurred in the northern coast of the Sea of Japan, L1b and L1c in the middle latitudes, and L1a in the southwest region (Fig 1; S4 Table). L1a—L1c haplotypes occurred also in Taiwan, and L1c in the coasts of China and Peninsular Malaysia (Fig 1). In Peninsular Malaysia, only one haplotype of L1c (H65; S4 Table) was detected, which was also found in Japan. Three laboratory colonies from Peninsular Malaysia, Thailand and Taiwan (not included in Fig 1) showed the same single L1c haplotype (H54), which was common in temperate Japan (S4 Table). Lineage L2 was represented by the only haplotype from Canadian populations, and it was found nowhere else (Fig 2). L2 was estimated to have diverged from L1 approximately 1 mya (95% HPD interval, 0.6–1.5 mya). L3 (7 haplotypes) was unique to the Ryukyu Islands from Amami to Yonaguni. Mosquitoes in the northernmost Ryukyu island, Amami also had L1a haplotypes, which were dominant in southern temperate Japan. Finally, a lineage L4 was found in east Hokkaido (6 haplotypes). The relationships between the geographic populations are depicted by the population tree based on mean sequence difference of COI sequences (Fig 3A); this tree shows that the populations from southern China, Taiwan and Peninsular Malaysia are closely related to populations in temperate Japan. The nucleotide diversity of COI sequences at each locality was high at middle latitude around 35°N and at a site on Amami Island (28.48°N) harboring both L1 and L3 haplotypes (Fig 3B).

**Fig 4:**
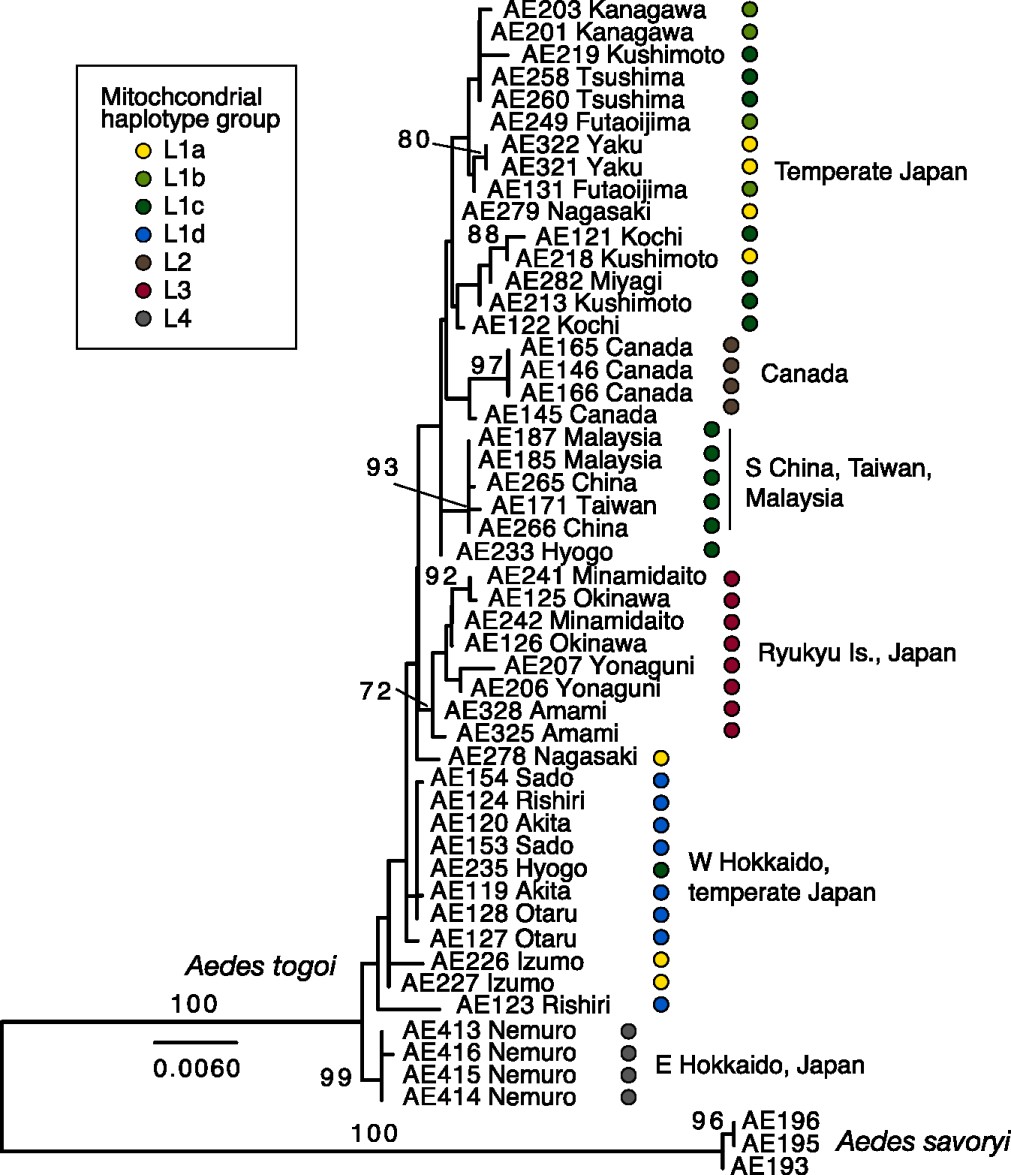
Maximum-likelihood tree of *Aedes togoi* resulting from combined analysis of three nuclear genes. Numerals on branches are bootstrap percentages (shown when >50%). Mitochondrial lineage of each individual is indicated.

We obtained 959-bp 28S, 819-bp CAD and 825-bp white gene sequences for 49 specimens of *A*. *togoi* (DDBJ accessions LC025729—LC025878). In the combined phylogenetic analysis of these nuclear gene sequences (Fig 4; see S3 Fig for individual gene trees), individuals with the same mitochondrial lineages tended to clump with one another although those with L1 haplotypes were polyphyletic. As in the mitochondrial gene tree, individuals with L4 mitochondrial haplotypes from eastern Hokkaido were distinct from all individuals with the other mitochondrial haplotypes in the nuclear gene trees. Canadian individuals showed unique substitutions in 28S and white genes, forming a monophyletic clade. Further, individuals from southern China, Taiwan and Peninsular Malaysia formed a strongly supported clade due to unique substitutions in CAD and white gene sequences. Substitutions in coding sequences of CAD and white genes were all synonymous.

## Discussion

The mitochondrial COI haplotypes of *A*. *togoi* consisted of four distinct lineages, which were estimated to have diverged since the late Pliocene or the early Pleistocene; the haplotype diversity and nucleotide diversity were highest in the temperate zone in Japan. The alternation of haplotype lineages across geographic and climatic regions in Japan, together with divergence in nuclear gene sequences corresponding to that in mitochondrial haplotypes, could be the effect of isolation by distance, but geographic disjunctions of haplotype lineages between temperate and subtropical or subarctic zones are consistent with the existence of geographic barriers, which may be associated with local adaptation.

Notably, the populations of the subtropical Ryukyu Islands of Japan had a haplotype lineage different from those in temperate Japan, suggesting the absence of continuous gene flow between these regions. Also notably, the population in Taiwan did not have the Ryukyu haplotypes despite their geographic proximity. In southern China, Taiwan and Peninsular Malaysia, *A*. *togoi* populations had only haplotypes of the L1 lineage, the same haplotypes as those found in temperate Japanese populations. These results suggest that the Southeast Asian populations have not been isolated long from those in temperate Japan. However, based on the nuclear gene sequences, the Southeast Asian populations formed a monophyletic group diverging from temperate Japanese populations. Therefore, the present Southeast Asian populations may have a common origin, either by natural or anthropogenic dispersal; a founder population might have subsequently dispersed while being adapted to subtropical and tropical environments. The dispersal of *A*. *togoi* might have been facilitated by transportation by ships among Asian countries, although direct evidence is lacking. During the mid-20th century, *Aedes aegypti* was introduced to Japan from Southeast Asia probably by ship; at this time *A*. *togoi* was common around the ports in Japan [40]. Also, Ramalingam [13] considered ships carrying cargo such as iron ore and timber between the east coast of Peninsular Malaysia and Japan as candidate transporters.

Although our genetic data suggest that the Southeast Asian populations of *A*. *togoi* have not been isolated very long from the temperate Japanese populations, the tropical populations from Thailand and Peninsular Malaysia had different life-history characteristics from those of temperate populations in the laboratory such as the lack of larval diapause, obligatory autogeny and small body size [19,21]. Another tropical population from Hainan I., China, also showed obligatory autogeny [41]. These differences may be evidence that the tropical populations are indigenous [19]. However, life history traits such as diapause and body size can evolve rapidly in dipteran insects including aedine mosquitoes [42–45]. Therefore, the life-history characteristics of *A*. *togoi* in the tropics may have evolved during their recent range expansion in East and Southeast Asia.

The origin of Canadian populations is enigmatic, as there is a gap of more than 5000-km in their distribution between East Asia and the North American Pacific coast. The fact that the Canadian population has life-history characteristics similar to temperate populations in Japan [19–20] does not contradict either of the hypotheses: anthropogenic introduction or natural distribution, because both climates are temperate. We found only a single, unique COI haplotype from four locations in British Columbia separated up to 150 km. One explanation may be that the Canadian population is an introduced one from somewhere in Asia, which started from few individuals or has experienced a strong bottleneck. Transportation by ship has been possible since the late 19th century, as there was already trade between Yokohama, Japan and Vancouver then. Mogi [46] noted that the underground mosquito *Culex pipiens* f. *molestus* might have been transported from North America to Japan by ship by the mid-20th century. However, we cannot exclude the possibility that *A*. *togoi* could have spread naturally across Beringia unless the origin of the unique haplotype is resolved by more extensive sampling in East Asia. In this hypothesis, *A*. *togoi* might have undergone stepping stone dispersal along the Kuril Islands, Kamchatka, the Aleutian Islands and Alaska to British Columbia. The estimated divergence time of the Canadian haplotype from L1 haplotypes in Asia was 1 mya (credible range, 0.6–1.5 mya), spanning several glacial cycles during which such dispersal across Beringia might have been possible. The last land bridge between Siberia and Alaska was not severed until just before 13,000 ya [47]. On the northeast Pacific coast, the easternmost occurrence of *A*. *togoi* is reported from South Kuril [7], close (<50 km) to eastern Hokkaido where the most diverged L4 haplotypes occurred. *Aedes togoi* may occur further east, on the North Kurils, Kamchatka, or the Aleutians; a survey of *A*. *togoi* in these regions is needed to understand the dispersal pattern of *A*. *togoi* in the subarctic zone and obtain a clue to the origin of the North American population.

Lastly, we note that, even though *A*. *togoi* can disperse over long distances by themselves or by human transportation, the survival of colonizers may be limited in exotic habitats, and their populations may often be subject to the loss of genetic diversity or extinction. For example, *A*. *togoi* once colonized the subtropical Ogasawara Islands in the Pacific Ocean 1000-km south of Tokyo in 1960s [48], probably as a result of transportation by ships. However, *A*. *togoi* now appears to be extinct, as only the native sister species *A*. *savoryi* was abundant in rock pools in later surveys (e.g., [49]; 2010 by K. Kawakami, personal communication). On the Ogasawara islands, reproductive interference with the sister species may make the survival of invasive species difficult [50–52]. Irrespective of the presence of closely related species, survival of introduced mosquito populations is limited by abiotic and biotic factors in exotic habitats [53]. Scarcity of large, stable rock pools on the seacoast would greatly limit the population persistence of *A*. *togoi*. On the eastern coast of Peninsular Malaysia, suitable larval habitats for *A*. *togoi* (rock pools or holes) are scarce, and the *A*. *togoi* population there appeared to be very small according to our field survey (in 1989 and 2010 by TS and HSY).

Our study revealed that the genetic diversity of *A*. *togoi* centers on the Japanese Islands, and the genetic composition varies among geographic and climatic regions, consistent with the existence of geographic barriers related to climatic adaptation as well as the effect of isolation-by-distance. The genetic composition of southern China, Taiwan and Southeast Asian populations is similar to those in temperate Japan, consistent with gene flow between these regions, whereas the Canadian population has a genetic composition distinct from that of Asia. Our study has not provided strong support for recent anthropogenic introduction of *A*. *togoi* from temperate Japan to tropical Southeast Asia or Canada, although transportation by ship may have promoted gene flow between Japan and other Asian countries. More extensive geographic sampling of *A*. *togoi* populations along the coast of East Asian mainland and around the Pacific rim will be needed to resolve the role of natural and anthropogenic dispersal in forming the present distribution of this species.

## Supporting Information

S1 Fig Phylogenetic relationships among culicine species showing the sister relationship between *Aedes togoi* and *A*. *savoryi*. The phylogenetic position of *A*. *togoi* and its sister relationship to *A*. *savoryi* based on a morphological analysis were examined using three nuclear gene sequences (CAD, enolase, white) newly obtained for *A*. *togoi* and *A*. *savoryi* with previously published data for 10 species from aedine and non-aedine Culicinae (S2 Table). The maximum-likelihood tree obtained by RAxML (partitioned by each codon position in each gene; GTR+gamma model; 1000 bootstrap analysis) weakly supported the monophyly of Aedini but strongly supported the sister relationship between *A*. *togoi* and *A*. *savoryi* within the strongly supported group of some aedine species.
(PDF)

S2 Fig Divergence time estimation by Bayesian relaxed clock analysis with BEAST. Median nodes age and 95% highest probability density intervals are indicated for major nodes with black circles. On the branches shown are posterior probabilities and bootstrap percentages in the maximum-likelihood analysis (shown when >0.5 or >50%).
(PDF)

S3 Fig Maximum-likelihood trees for three nuclear genes. Bootstrap percentages are shown on the branches when >50%.
(PDF)

S1 Table List of samples for DNA analysis with information of haplotype groups. (PDF)

S2 Table GenBank/DDBJ accession numbers for the three genes (exon only) used in the phylogenetic analysis of culicine mosquitoes. (PDF)

S3 Table Mean sequence divergence (uncorrected p) and estimated divergence times between mitochondrial COI haplotype lineages. (PDF)

S4 Table Distribution of mitochondrial COI haplotypes. (PDF)

## References

[1] Tabachnick WJ, Powell JR. A world-wide survey of genetic variation in the yellow fever mosquito, *Aedes aegypti* . Genet Res Camb. 1979;34: 215–229. 10.1017/s0016672300019467 544311

[2] Hawley WA, Reiter P, Copeland R S, Pumpuni CB, Craig GB. *Aedes albopictus* in North America—probable introduction in used tires from northern Asia. Science 1987;236: 1114–1116. 3576225 10.1126/science.3576225

[3] Kambhampati S, Black WC IV, Rai KS. Geographic origin of the US and Brazilian *Aedes albopictus* inferred from allozyme analysis. Heredity 1991;67: 85–94. 1917554 10.1038/hdy.1991.67

[4] Mousson L, Dauga C, Garrigues T, Schaffner F, Vazeille M, Failloux A. Phylogeography of *Aedes* (*Stegomyia*) *aegypti* (L.) and *Aedes* (*Stegomyia*) *albopictus* (Skuse) (Diptera: Culicidae) based on mitochondrial DNA variations. Genet Res Camb. 2005;86: 1–11. 10.1017/S0016672305007627 16181519

[5] Kaufman MG, Fonseca DM. Invasion biology of *Aedes japonicus japonicus* . Annu Rev Entomol. 2014;59: 31–49. 10.1146/annurev-ento-011613-162012 24397520 PMC4106299

[6] Kampen H, Werner D. Out of the bush: the Asian bush mosquito *Aedes japonicus japonicus* (Theobald, 1901) (Diptera, Culicidae) becomes invasive. Parasites Vectors. 2014;7:59 10.1186/1756-3305-7-59 24495418 PMC3917540

[7] Tanaka K, Mizusawa K, Saugstad ES. A revision of the adult and larval mosquitoes of Japan (including the Ryukyu Archipelago and the Ogasawara Islands) and Korea. Contr Am Entomol Inst 1979;16: 1–987.

[8] Rosen L. The natural history of Japanese encephalitis virus. Annu Rev Microbiol. 1986;40: 395–414. 2877613 10.1146/annurev.mi.40.100186.002143

[9] Cheun HI, Cho SH, Lee HI, Shin EH, Lee JS, Kim TS, et al Seasonal prevalence of mosquitoes, including vectors of Brugian filariasis, in southern islands of the Republic of Korea. Korean J Parasitol. 2011;49: 59–64. 10.3347/kjp.2011.49.1.59 21461270 PMC3063927

[10] Cho SH, Ma DW, Koo BR, Shin HE, Lee WK, Jeong BS, et al Surveillance and vector control of lymphatic filariasis in the Republic of Korea. OSong Public Health Res Perspect. 2012;3: 145–150. 10.1016/j.phrp.2012.07.008 24159506 PMC3738707

[11] Quy DV. Entomological surveys in the islands of Hon-Yen and Phu-Quy. Les Exped Sci. 1966; 1:89–98.

[12] Gould DJ, Yuill TM, Moussa MA, Simatien P, Rutledge LC. An insular epidemic outbreak of dengue haemorrhagic fever III. Identification of vectors and observation on vector ecology. Am J Trop Med Hyg. 1968;17: 609–618. 5672791 10.4269/ajtmh.1968.17.609

[13] Ramalingam S. New record of *Aedes* (*Finlaya*) *togoi* (Theobold) in West Malaysia. Med J Malaya. 1969;23: 288–292. 4242176

[14] Wood DM, Dang PT, Ellis RA. The mosquitoes of Canada. Diptera: Culicidae. The insects and arachnids of Canada. Part 6. Ministry of Supply and Service of Canada, Quebec; 1979.

[15] Belton P (1980) The first record of *Aedes togoi* (Theo.) in the United States—aboriginal or ferry passenger? Mosq News 40:624–626.

[16] Belton P, Belton OC. *Aedes togoi* comes aboard. J Am Mosq Control Assoc. 1990;6: 328–329. 2370542

[17] Mogi M. Studies on *Aedes togoi* (Diptera: Culicidae) 1. Alternative diapause in the Nagasaki strain. J Med Entomol. 1981;18: 477–480.

[18] Sota T, Mogi M. Seasonal life cycle and autogeny in the mosquito *Aedes togoi* in northern Kyushu, Japan, with experimental analysis of the effects of temperature, photoperiod and food on life-history traits. Res Popul Ecol. 1994;36: 105–114.

[19] Mori A, Buei K, Phan-Urai P, Fujita K. Difference in biological characteristics between *Aedes togoi* originated from Thailand and Japan. Trop Med (Nagasaki) 1985;27: 283–288.

[20] Sota T. Larval diapause, size, and autogeny in the mosquito *Aedes togoi* (Diptera, Culicidae) from tropical to subarctic zones. Can J Zool. 1994;72: 1462–1468.

[21] Sota T, Mogi M. Geographic variation in the expression of autogeny in *Aedes togoi* (Diptera: Culicidae) under different temperature and photoperiod conditions. J Med Entomol. 1995;32: 181–189. 7608925 10.1093/jmedent/32.2.181

[22] Mogi M, Okazawa T, Sota T. Geographical pattern in autogeny and wing length in *Aedes togoi* (Diptera: Culicidae). Mosq Syst. 1995;27:155–166.

[23] Galka BE, Brust RA. The effect of temperature and photoperiod on the induction of larval diapause in the mosquito *Aedes togoi* (Theobald) (Diptera: Culicidae). Can J Zool. 1987;65: 2262–2265.

[24] Galka BE, Brust RA. The effect of temperature and photoperiod on the induction of embryonic diapause in the mosquito *Aedes togoi* (Theobald) (Diptera: Culicidae). Can J Zool 1987;65: 2266–2271.

[25] Reinert JF, Harbach RR, Kitching IJ. Phylogeny and classification of Aedeni (Diptera: Culicidae), based on morphological characters of all life stages. Zool J Linn Soc. 2004;142: 289–368.

[26] Reidenbach KR, Cook S, Bertone MA, Harbach RE, Wiegmann BM, Besansky NJ. Phylogenetic analysis and temporal diversification of mosquitoes (Diptera: Culicidae) based on nuclear genes and morphology. BMC Evol Biol. 2009;9:298 10.1186/1471-2148-9-298 20028549 PMC2805638

[27] Simon C, Frati F, Beckenbach A, Crespi B, Liu H, Flook P. Evolution, weighting, and phylogenetic utility of mitochondrial gene sequences and a compilation of conserved polymerase chain reaction primers. Ann Entomol Soc Am. 1994;87: 651–701.

[28] Sota T, Mogi M. Origin of pitcher plant mosquitoes in *Aedes* (*Stegomyia*): a molecular phylogenetic analysis using mitochondrial and nuclear gene sequences. J Med Entomol. 2006;43: 795–800. 17017211 10.1603/0022-2585(2006)43[795:ooppmi]2.0.co;2

[29] Edgar RC. MUSCLE: multiple sequence alignment with high accuracy and high throughput. Nuc Acid Res. 2004;32: 1792–1797. 10.1093/nar/gkh340 PMC390337 15034147

[30] Stamatakis A. RAxML Version 8: a tool for phylogenetic analysis and post-analysis of large phylogenies. Bioinformatics 2014; 10.1093/bioinformatics/btu033 PMC3998144 24451623

[31] Huson DH, Bryant D. Application of phylogenetic networks in evolutionary studies. Mol Biol Evol. 2006;23:254–267. 16221896 10.1093/molbev/msj030

[32] Tamura K, Peterson D, Peterson N, Stecher G, Nei M, Kumar S. MEGA5: Molecular evolutionary genetics analysis using maximum likelihood, evolutionary distance, and maximum parsimony methods. Mol Biol Evol. 2011;28: 2731–2739. 10.1093/molbev/msr121 21546353 PMC3203626

[33] Felsenstein J. PHYLIP (Phylogeny Inference Package) version 3.6. Distributed by the author Department of Genome Sciences, University of Washington, Seattle; 2005.

[34] Excoffier L, Lischer HEL. Arlequin suite ver 3.5: A new series of programs to perform population genetics analyses under Linux and Windows. Mol Ecol Res. 2010;10: 564–567. 10.1111/j.1755-0998.2010.02847.x 21565059

[35] Drummond AJ, Suchard MA, Xie D, Rambaut A. Bayesian phylogenetics with BEAUti and the BEAST 1.7. Mol Biol Evol. 2012;29: 1969–1973 10.1093/molbev/mss075 22367748 PMC3408070

[36] Watanabe K, Takahashi H, Kitamura A, Yokoyama R, Kitagawa T, Takeshima H, Sato S, Yamamoto S, Takehana Y, Mukai T, Ohara K, Iguchi K. Biogeographical history of Japanese freshwater fishes: phylogeographic approaches and perspectives. Jap J Ichthyol. 2006;53: 1–38.

[37] Rambaut A, Suchard MA, Xie D, Drummond AJ. Tracer v1.6;2014. Available: http://beast.bio.ed.ac.uk/Tracer.

[38] Ho SYW, Phillips MJ, Cooper A, Drummond AJ. Time dependency of molecular rate estimates and systematic overestimation of recent divergence times. Mol Biol Evol. 2005;22: 1561–1568. 15814826 10.1093/molbev/msi145

[39] Papadopoulou A, Anastasiou I, Vogler AP. Revisiting the insect mitochondrial molecular clock: the mid-Aegean trench calibration. Mol Biol Evol. 2010;27: 1659–1672. 10.1093/molbev/msq051 20167609

[40] Kurihara T. Review of dengue vector mosquitoes in Japan. Med Entomol Zool. 2003;54: 135–154.

[41] Sota T, Wang N, Huang Z-J. Autogeny of *Aedes togoi* (Diptera, Culicidae) from Hainan, southern China. Jpn J Sanit Zool. 1995;46: 173–175.

[42] Huey RB, Gilchrist GW, Carlson ML, Berrigan D, Serra L. Rapid evolution of a geographic cline in size in an introduced fly. Science 2000;287: 308–309. 10634786 10.1126/science.287.5451.308

[43] Gilchrist GW, Huey RB, Serra L. Rapid evolution of wing size clines in *Drosophila subobscura* . Genetica 2001;112–113: 273–286. 11838770

[44] Lounibos LP, Escher RL, Lourenco-de-Oliverira R. Asymmetric evolution of photoperiodic diapause in temperate and tropical invasive populations of *Aedes albopictus* (Diptera: Culicidae). Ann Entomol Soc Am. 2003;96: 512–518.

[45] Urbanski J, Mogi M, O'Donnell D, DeCotiis M, Toma T, Armbruster P. Rapid adaptive evolution of photoperiodic response during invasion and range expansion across a climatic gradient. Am Nat. 2012;179: 490–500. 10.1086/664709 22437178

[46] Mogi M. The forms of the *Culex pipiens* complex in East Asia, with ecological thoughts on their origin and interrelation. J Am Mosq Control Assoc. 2012;28(4 suppl): 28–52. 23401943 10.2987/8756-971X-28.4s.28

[47] Matthews JV. Historical background for an analysis of the Canadian insect fauna. In: Danks HV, editor. Canada and its insect Fauna. Mem Ent Soc Can. 1979;108:65

[48] Takahashi S. Insects of medical importance in Ogasawara (Bonin) Islands. Jap J Sanit Zool. 1973;24: 143–148. (In Japanese with English summary)

[49] Toma T, Miyagi I. Notes on mosquitoes in Chichi-jima, Ogasawara Archipelago, Japan and biology of *Culex* (*Sirivanakarnius*) *boninensis* (Diptera: Culicidae). Med Entomol Zool. 2005;56: 237–241.

[50] Ribeiro JMC, Spielman A. The satyr effect: a model predicting parapatry and species extinction. Am Nat. 1986;128:513–528.

[51] Kuno E. Competitive exclusion through reproductive interference. Res Popul Ecol. 1992;34:275–284

[52] Tripet F, Lounibos LP, Robbins D, Moran J, Nishimura N, Blosser EM. Competitive reduction by Satyrization? Evidence for interspecific mating in nature and asymmetric reproductive competition between invasive mosquito vectors. Am J Trop Med Hyg. 2011;85: 265–270. 10.4269/ajtmh.2011.10-0677 21813845 PMC3144823

[53] Juliano SA, Lounibos LP. Ecology of invasive mosquitoes: effects on resident species and on human health. Ecol Lett. 2005;8: 558–574. 17637849 10.1111/j.1461-0248.2005.00755 PMC1920178

