# Supplementary material for: Phylogeography of the Coastal Mosquito Aedes togoi across Climatic Zones: Testing an Anthropogenic Dispersal Hypothesis
Source: PLoS One. 2015 Jun 24;10(6):e0131230. doi: 10.1371/journal.pone.0131230 (PMC4479490; doi:10.1371/journal.pone.0131230)
Supplement: S1 Fig — The phylogenetic position of A. togoi and its sister relationship to A. savoryi based on a morphological analysis were examined using three nuclear gene sequences (CAD, enolase, white) newly obtained for A. togoi and A. savoryi with previously published data for 10 species from aedine and non-aedine Culicinae (S2 Table). The maximum-likelihood tree obtained by RAxML (partitioned by each codon position in each gene; GTR+gamma model; 1000 bootstrap analysis) weakly supported the monophyly of Aedini but strongly supported the sister relationship between A. togoi and A. savoryi within the strongly supported group of some aedine species. (PDF) [file pone.0131230.s001.pdf]

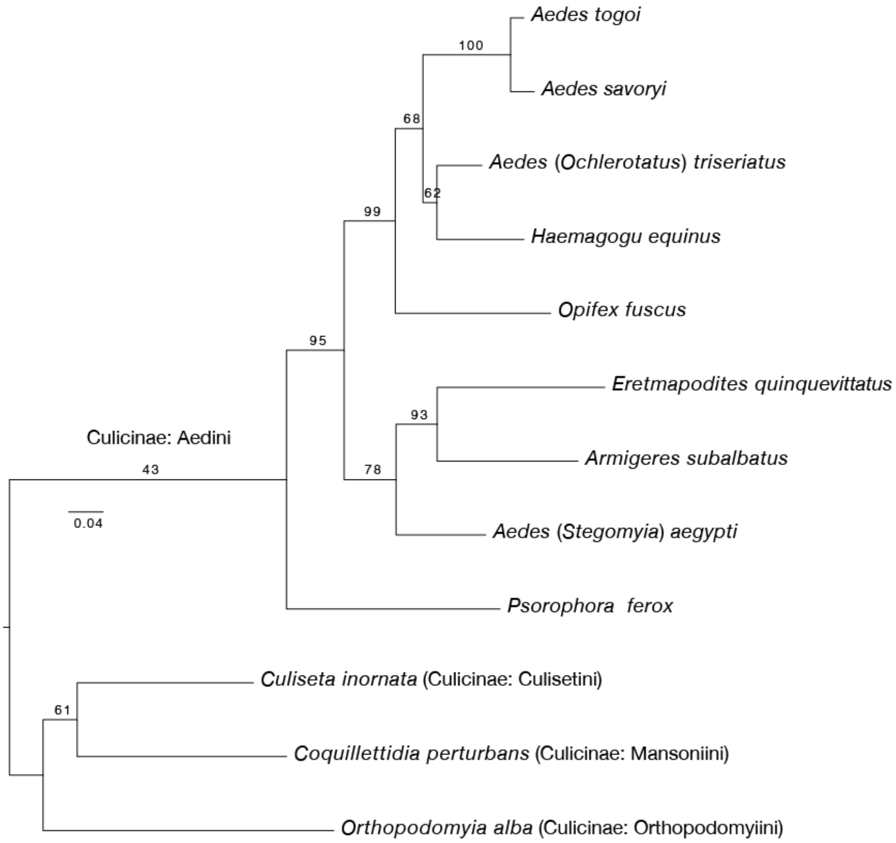

S1 Fig. Phylogenetic relationships among culicine species showing the sister relationship between *Aedes togoi* and *A. savoryi*. The phylogenetic position of *A. togoi* and its sister relationship to *A. savoryi* based on a morphological analysis were examined using three nuclear gene sequences (CAD, enolase, white) newly obtained for *A. togoi* and *A. savoryi* with previously published data for 10 species from aedine and non-aedine Culicinae (Table S2). The maximum-likelihood tree obtained by RAxML (partitioned by each codon position in each gene; GTR+gamma model; 1000 bootstrap analysis) weakly supported the monophyly of Aedini but strongly supported the sister relationship between *A. togoi* and *A. savoryi* within the strongly supported group of some aedine species.
