# Supplementary material for: Phylogeography of the Coastal Mosquito Aedes togoi across Climatic Zones: Testing an Anthropogenic Dispersal Hypothesis
Source: PLoS One. 2015 Jun 24;10(6):e0131230. doi: 10.1371/journal.pone.0131230 (PMC4479490; doi:10.1371/journal.pone.0131230)
Supplement: S2 Fig — Median nodes age and 95% highest probability density intervals are indicated for major nodes with black circles. On the branches shown are posterior probabilities and bootstrap percentages in the maximum-likelihood analysis (shown when >0.5 or >50%). (PDF) [file pone.0131230.s002.pdf]

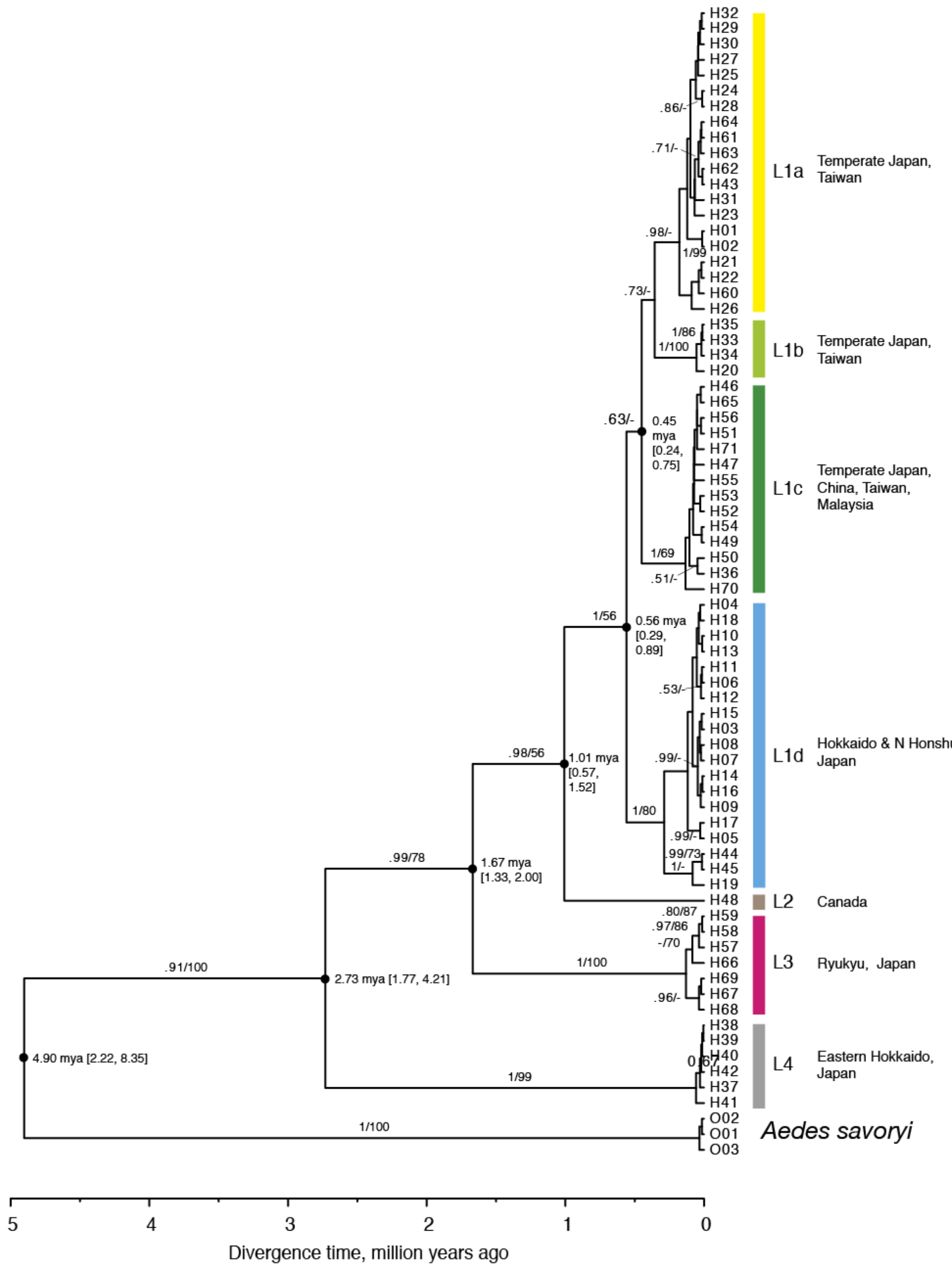

S2 Fig. Divergence time estimation by Bayesian relaxed clock analysis with BEAST. Median nodes age and 95% highest probability density intervals are indicated for major nodes with black circles. On the branches shown are posterior probabilities and bootstrap percentages in the maximum-likelihood analysis (shown when >0.5 or >50%).
