# Supplementary material for: Phylogeography of the Coastal Mosquito Aedes togoi across Climatic Zones: Testing an Anthropogenic Dispersal Hypothesis
Source: PLoS One. 2015 Jun 24;10(6):e0131230. doi: 10.1371/journal.pone.0131230 (PMC4479490; doi:10.1371/journal.pone.0131230)
Supplement: S3 Fig — Bootstrap percentages are shown on the branches when >50%. (PDF) [file pone.0131230.s003.pdf]

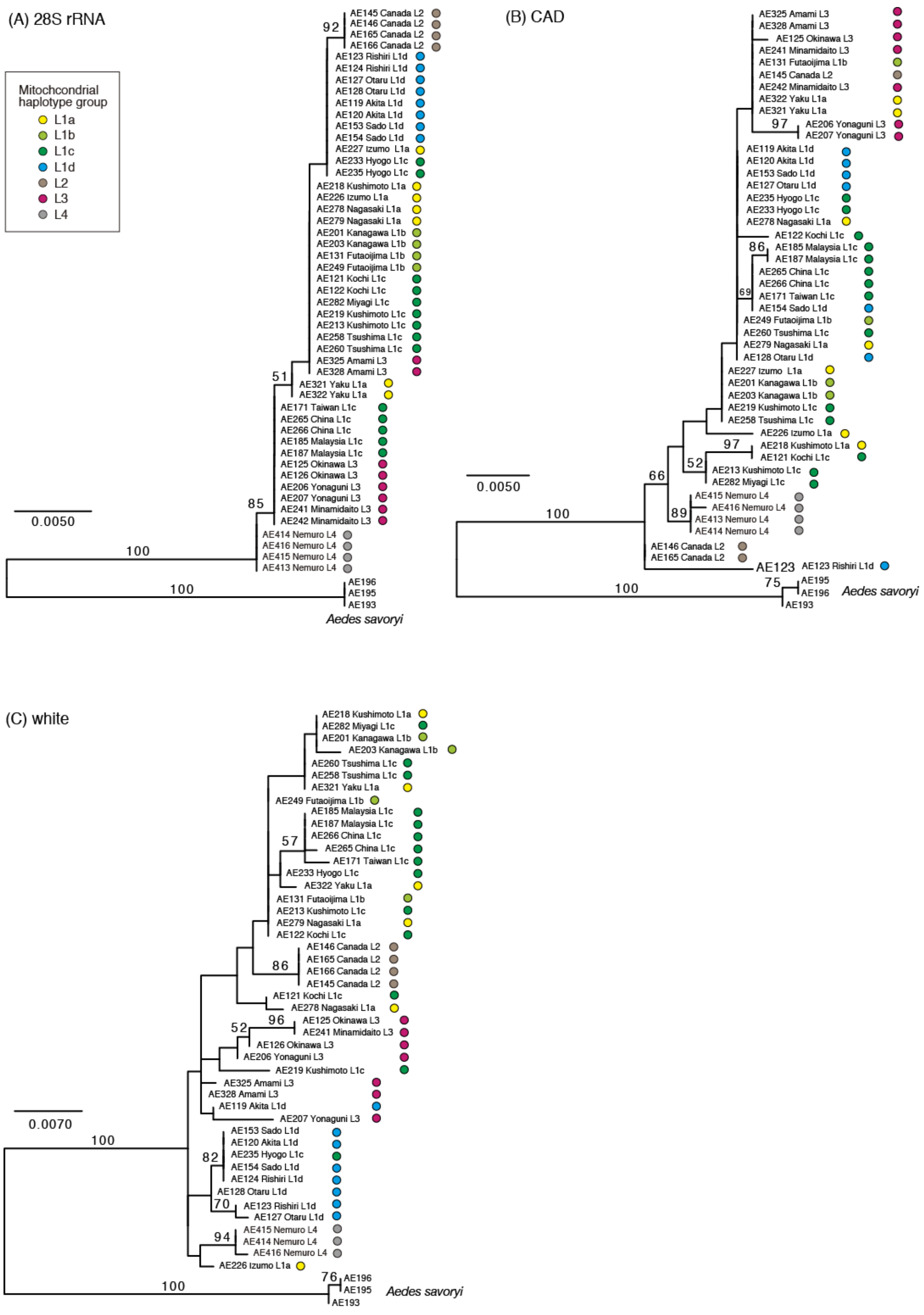

S3 Fig. Maximum-likelihood trees for three nuclear genes. Bootstrap percentages are shown on the branches when >50%.
