# Supplementary material for: Phylogeography of the Coastal Mosquito Aedes togoi across Climatic Zones: Testing an Anthropogenic Dispersal Hypothesis
Source: PLoS One. 2015 Jun 24;10(6):e0131230. doi: 10.1371/journal.pone.0131230 (PMC4479490; doi:10.1371/journal.pone.0131230)
Supplement: S1 Table — (PDF) [file pone.0131230.s004.pdf]

S1 Table. List of samples for DNA analysis with information of haplotype groups.

Locality code	Country	Locality	Year	Collector	*n*	Latitude, °N	Longitude, °E (°W for Canada)
Aedes togoi							
01_Rishiri	Japan	Rishiri I., Hokkaido	2008	M. Sato	8	45.1069	141.0202
02_Nosappu	Japan	Nemuro Peninsula., Hokkaido	2014	T. Sota	12	43.3382	145.7518
02_Otaru	Japan	Otaru, Hokkaido	2008	M. Mogi	6	43.2380	141.0159
03_Aomori	Japan	Utozaki, Aomori	2008	Y. Okuzaki	4	40.8830	140.8504
04_Akita	Japan	Oga Peninsula, Akita	2009	S. Yamamoto	4	39.8942	139.8575
05_Sado	Japan	Tassha, Sado I., Niigata	2009	J. Konuma	4	38.0754	138.2439
06_Fukui	Japan	Tojinbo, Fukui	2013	N. Nagata	3	36.2372	136.1256
07_Hyogo	Japan	Shinonsen, Hyogo	2009	T. Sota	8	35.6214	134.4242
08_Oki	Japan	Dogo I., Oki Is., Shimane	2009	T. Sota	2	32.9583	130.2199
09_Matsue	Japan	Mitsu, Matsue, Shimane	2009	T. Sota	4	35.5403	133.0241
10_Izumo	Japan	Tagi, Izumo, Shimane	2009	T. Sota	8	35.2753	132.5862
11_Miyagi	Japan	Sabusawa I, Shiogama, Miyagi	2012	N. Nagata	16	38.3331	141.1254
12_Kanagawa	Japan	Yokosuka, Kanagawa	2009	N. Nagata	12	35.2953	139.6791
12_KanagawaJ	Japan	Jogashima, Kanagawa	2014	H. Sugawara	8	35.1300	139.6278
12b_Aichi	Japan	Takeshima, Aichi	2014	M. Hoso	3	34.8102	137.2316
13_Kushimoto	Japan	Kushimoto, Wakayama	2010	K. Tsuji	8	33.4992	135.7990
14_Shirahama	Japan	Shirahama, Wakayama	2010	T. Sota	4	33.6669	135.3329
15_Kochi	Japan	Ashizuri-misaki, Kochi	2009	T. Sota	3	32.7241	133.0202
16_Tsushima	Japan	Tsutsuzaki, Tsushima I., Nagasaki	2010	Y. Okuzaki	7	34.1021	129.1681
17_Futaoijima	Japan	Futaoijima I., Yamagushi	2008	M. Mogi	8	34.1007	130.7881
18_Nagasaki	Japan	Aikawa, Nagasaki	2011	Y. Okuzaki	8	32.7939	129.7822
19_Amakusa	Japan	Shimoda, Amakusa, Kumamoto	2010	Y. Okuzaki	2	32.4279	130.0069
19_Koshiki	Japan	Nakakoshiki I. Koshiki Is., Kagoshima	2011	Y. Okuzaki	4	31.8221	129.8407
20_SagaN	Japan	Nanatsugama, Saga	2008	M. Mogi	3	33.5498	129.9322
21_SagaT	Japan	Takesaki, Saga	2005	M. Mogi	4	32.9583	130.2199
22_Fukuoka	Japan	Itoshima, Fukuoka	2011	Y. Okuzaki	4	33.5829	130.0888
23_Ohita	Japan	Saeki, Ohita	2011	Y. Okuzaki	4	32.9602	131.9606
24_Tane	Japan	Tanegashima I., Kagoshima	2008	M. Mogi	4	30.6677	130.9411
25_Yaku	Japan	Miyanoura, Yakushima I., Kagoshima	2009	N. Nagata	3	30.4340	130.5671
25_Yaku	Japan	Isso, Yakushima I., Kagoshima	2008	T. Sota	2	30.4581	130.5005

26_AmamiA	Japan	Ankyaba, Amami-oshima I., Kagoshima	2009	K. Tsuji	7	28.4757	129.6205
26_AmamiK	Japan	Kasari, Amami-oshima I., Kagoshima	2009	K. Tsuji	8	28.4608	129.6750
27_Okinawa	Japan	Gushikami, Okinawa I., Okinawa	2008	M. Mogi	7	26.1214	127.7555
28_Minamidaito	Japan	Minamidaitojima I., Okinawa	2009	M. Toda	7	25.8324	131.2670
29_Yonaguni	Japan	Yonaguni I., Okinawa	2010	T. Yashiro	7	24.4498	122.9348
30_Shenzhen	China	Shenzhen, China	2011	H. Liang/T. Sota	16	22.5443	114.6036
31_Taiwan	Taiwan	Iyeliu, Taiwan	2010	T. Sota	17	25.2075	121.6922
32_TaiwanLab	Taiwan	Laboratory strain (Mahidol University)	2009	C. Apiwathnasorn	4	-	-
33_ThaiLab	Thai	Laboratory strain (Chiang Mai University); origin, Koh Nom Sao, Chantaburi, 1981.	2009	W. Choochote	4	-	-
34_Malaysia	Malaysia	Pantai Kemasik, Malaysia	2010	H.S. Yong/T, Sota	16	4.4155	100.4567
35_MalaysiaLab	Malaysia	Laboratory strain (Institute of Medical Research)	2000	A. Mori	3	-	-
36_Canada	Canada	Horseshoe Bay, Vancouver	2009	M. Tseng	4	49.3701	123.2925
36_Canada	Canada	10 Mile Point, north and east of Victoria in the district of Oak Bay, Vancouver I. (I5)	2009	R. B. Bennett	4	48.4561	123.2689
36_Canada	Canada	Point Atkinson, Lighthouse Park, Vancouver (M4)	2009	P. Belton	4	49.8300	123.2646
36_Canada	Canada	Helby Island, West Coast of Vancouver Island, British Columbia	2010	J. G. Taylor	4	48.8500	125.1667
Aedes sevoryi	Japan	Suzaki, Chichijima I., Tokyo	2010	K. Kawakami	8	27.0893	142.1876
