# Supplementary material for: Phylogeography of the Coastal Mosquito Aedes togoi across Climatic Zones: Testing an Anthropogenic Dispersal Hypothesis
Source: PLoS One. 2015 Jun 24;10(6):e0131230. doi: 10.1371/journal.pone.0131230 (PMC4479490; doi:10.1371/journal.pone.0131230)
Supplement: S2 Table — (PDF) [file pone.0131230.s005.pdf]

**S2 Table. GenBank/DDBJ accession numbers for the three genes (exon only) used in the phylogenetic analysis of culicine mosquitoes.**

| Species | | | |
|---|---|---|---|
| Gene | CAD | enolase | white |
| aligned bp | 819 | 625 | 378 |
| *Culiseta inornata* | GQ906834 | GQ906882 | GQ906928 |
| *Orthopodomyia alba* | GQ906844 | GQ906892 | AF318206 |
| *Coquillettidia perturbans* | GQ906835 | GQ906883 | n.a. |
| *Psorophora ferox* | GQ906845 | GQ906893 | GQ906935 |
| *Aedes* (*Stegomyia*) *aegypti* | n.a | XM_001653700 | U73826 |
| *Eretmapodites quinquevittatus* | GQ906836 | GQ906884 | GQ906929 |
| *Armigeres subalbatus* | GQ906832 | n.a. | AF318200 |
| *Opifex fuscus* | GQ906843 | GQ906891 | GQ906934 |
| *Haemagogus equinus* | GQ906837 | GQ906885 | U73834 |
| *Ochlerotatus triseriatus* | GQ906842 | GQ906890 | U73827 |
| | | | |
| *Aedes togoi* | LC025781 | LC026054 | LC025830 |
| *Aedes savoryi* | LC025795 | LC026055 | LC025844 |

Note: Although *A. togoi* and *A. savoryi* are classified into the genus *Tanakaius* (Reinert et al. 2004), we retain to use *Aedes* for these species; other species names follow Reidenbach et al. (2009).
