# Supplementary material for: Phylogeography of the Coastal Mosquito Aedes togoi across Climatic Zones: Testing an Anthropogenic Dispersal Hypothesis
Source: PLoS One. 2015 Jun 24;10(6):e0131230. doi: 10.1371/journal.pone.0131230 (PMC4479490; doi:10.1371/journal.pone.0131230)
Supplement: S3 Table — (PDF) [file pone.0131230.s006.pdf]

S3 Table. Mean sequence divergence (uncorrected p) and estimated divergence times between mitochondrial COI haplotype lineages.

Lineages	Mean sequence divergence (± SD)	Divergence time in million years
L1 - L2	0.0296 ± 0.0035	0.62
L1 - L3	0.0446 ± 0.0068	1.20
L1 - L4	0.0598 ± 0.0027	2.03
L2 - L3	0.0504 ± 0.0009	1.49
L2 - L4	0.0577 ± 0.0018	1.91
L3 - L4	0.0653 ± 0.0023	2.38

The divergence times between COI haplotype lineages were estimated based on uncorrected p distances and an insect mitochondrial molecular clock with time dependent evolutionary rate (Papadopoulou et al. 2010). We used an equation in Ho et al. (2005; eq. 7) to obtain divergence time from given sequence divergence by numerical analysis. Sequence divergence d is expressed as: $d = 1/\lambda\ (-\mu e^{-\lambda t} + k\lambda t + \mu)$, where λ is a constant inversely proportional to the half-life of the rate decay, μ is the instantaneous mutation rate, t is the divergence date of two sequences, and k is a finite asymptote that represents the evolutionary rate over long time periods. Papadopoulou et al. (2010) estimated $\mu = 0.054$, $\lambda = 1.794$, and $k = 0.015$ for a set of mitochondrial gene sequence divergence data in insects.
