# Supplementary material for: Phylogeography of the Coastal Mosquito Aedes togoi across Climatic Zones: Testing an Anthropogenic Dispersal Hypothesis
Source: PLoS One. 2015 Jun 24;10(6):e0131230. doi: 10.1371/journal.pone.0131230 (PMC4479490; doi:10.1371/journal.pone.0131230)
Supplement: S4 Table — (PDF) [file pone.0131230.s007.pdf]

S4 Table. Distribution of mitochondrial COI haplotypes.

Lineage	L1a																					L1b					L1c															L1d																				L2		L3								L4							Total
Haplotype	H1	H2	H21	H22	H23	H24	H25	H26	H27	H28	H29	H30	H31	H32	H43	H60	H61	H62	H63	H64	Total	H20	H33	H34	H35	Total	H36	H46	H47	H49	H50	H51	H52	H53	H54	H55	H56	H65	H70	H71	Total	H3	H4	H5	H6	H7	H8	H9	H10	H11	H12	H13	H14	H15	H16	H17	H18	H19	H44	H45	Total	H48	Total	H57	H58	H59	H66	H67	H68	H69	Total	H37	H38	H39	H40	H41	H42	Total	
01_Rishiri																																										1				1	1	1					2	1	1						8																		8
02_Nosappu																																																																								1	5	2	1	1	2	12	12
02_Otaru																																																		2		1	2					1			6																		6
03_Aomori																															4										4																																						4
04_Akita																																											1								1						2				4																		4
05_Sado																																													1															3	4																		4
06_Fukui											1										1																							1					1												2																		3
07_Hyogo								1	1		2			1							5														1			1			2															1					1																		8
08_Oki							1								1						2																																																											2
09_Matsue											1										1														1						1																		1	1	2																		4
10_Izumo											2							1			3								1		1		2								4																			1	1																		8
11_Miyagi																							1		1	2			1		11				2						14																																						16
12_Kanagawa																						12				12																																																							12
12_KanagawaJ																		2			2														6						6																																						8
12b_Aichi																			2		2														1						1																																						3
13_Kushimoto																		3			3										3				2						5																																						8
14_Shirahama																		2			2										1				1						2																																						4
15_Kochi																															2			1							3																																						3
16_Tsushima																															2				2	1			2		7																																						7
17_Futaoijima																						2				2									6						6																																						8
18_Nagasaki							1											2		1	4								1								2			1	4																																						8
19_Amakusa										1			1								2																																																										2
19_Koshiki	1						1											2			4																																																										4
20_SagaN										2											2														1						1																																						3
21_SagaT		3																			3										1										1																																						4
22_Fukuoka						1											2				3									1											1																																						4
23_Ohita																		3			3								1												1																																						4
24_Tane					1		1					1						1			4																																																										4
25_Yaku			1	1			3														5																																																									5
26_AmamiA							1									4					5																																														2			2								7
26_AmamiK																																																																			8			8								8
27_Okinawa																																																																	1			6			7								7
28_Minamidaito																																																																5		2					7								7
29_Yonaguni																																																																			4		1	2	7								7
30_Shenzhen																												1				1			14						16																																						16
31_Taiwan																		1			1			1		1	1		3		3				8						15																																						17
32_TaiwanLab																																			4						4																																						4
33_ThaiLab																																			4						4																																						4
34_Malaysia																																						16			16																																						16
35_MalaysiaLab																																			3						3																																						3
36_Canada																																																													16	16																16
Total	1	3	1	1	1	1	8	1	1	3	6	1	1	1	1	4	2	17	2	1	57	14	1	1	1	17	1	1	7	1	28	1	2	1	56	1	2	17	2	1	121	1	1	1	1	1	1	1	1	2	1	1	4	1	1	1	2	1	1	5	28	16	16	5	1	2	4	16	1	2	31	1	5	2	1	1	2	12	282
